# The Differential Diagnosis of the Cause of Sudden Unconsciousness

**Published:** 1882-06

**Authors:** R. O. Beard


CHICAGO MEDICAL

JOURNAL & EXAMINER.

Vol. XLIV.—JUNE, 1882.—No. 6.

### Original Communications.

Article I.

### The Differential Diagnosis of the Cause of Sudden Unconsciousness. By R. O. Beard, M. D.

It is a matter of wonder that to a subject of such grave importance, as "The Differential Diagnosis of the Cause of Sudden Unconsciousness," so little distinctive attention has been paid, for though unconsciousness be but a *symptom* of diseased or perverted action, it is one of such vital consequence, often one of such imminent danger, that a fortunate or fatal issue frequently turns upon the pivot of the prompt diagnosis and intelligent care of the physician. Even granting that in any given case all idea of an immediately fatal tendency is eliminated,—supposing that the conditions point to nothing more serious than ordinary inebriation—it must not be forgotten that upon the doctor's diagnosis and the expression of his opinion may hinge not only his own reputation, but also the moral character of a, perhaps, hitherto innocent individual.

Mistakes of this nature have been made, even by reputable practitioners, and more than one victim has in consequence, suffered death in forced confinement, without care or treatment, and found vindication only in an autopsy which revealed an organic lesion as the cause, first of coma, and subsequently of decease.

The need of a careful study of the various causes productive of this phenomenon is thus prominent, because their differentiation is apt to be so obscure that physicians of undoubted ability have found themselves baffled.

In this paper I propose to consider only those conditions in which unconsciousness is of sudden occurrence, unattended by marked or continued prodromata, and sufficiently complete to render the patient incapable of furnishing subjective evidence or prior history. I shall endeavor to outline the distinctive symptoms observable in these cases and as the readiest means of studying these I shall append a summarized table which may prove of some diagnostic value.

The possibility of the co-existence of two or more diseases giving rise to this condition, or of the complication of disease with accident, or *vice versa* must always be born in mind. Apoplexy or sunstroke may supervene in a state of acute alcoholism; cerebral congestion or embolism may be accompanied by slight hæmorrhage. An apoplectic attack has been known to follow an epileptic seizure, and contusion, concussion and compression of the brain may be combined as results of severe injury.

Under the generic term of *Apoplexy*, a relic of the old-fashioned nomenclature, is grouped a class of cases almost involving coma, but with variations sufficiently distinct to indicate the differing seat and character of the causative lesion. These are, (1) cerebral congestion, (2) cerebral hæmorrhage and (3) meningeal hæmorrhage. In

CEREBRAL CONGESTION

unconsciousness is rarely of primary occurrence. It is usually preceded by general hyperæmic symptoms, and only in occasional cases is the initial evidence of disturbed function. Its distinctive features are: a partial paralysis, frequently bilateral but rarely, if ever, hemiplegic, contracted pupils with feeble reaction, temperature continuously higher than normal, respiration slow and labored but lacking the stertor and peculiar expiratory puffing of the lips and cheeks observed in cerebral hæmorrhage, venous distention of the face and neck, and the comparatively rapid restoration of mental and muscular power.

This condition is not unfrequently superinduced by sunstroke or alcoholic stimulation, and if prolonged may end in serous effusion and death.

The existence or non-existence of hypertrophy of the heart, as an exciting cause, should be determined, and may serve as a valuable aid in diagnosis.

The opinion held by Trousseau that so-called apoplectiform congestion is always epileptic in character is certainly untenable, not only because many of the general symptoms are essentially different, but also because an epileptic attack in which coma is as prolonged as it is in many cases of cerebral congestion must constitute the graver form of the disease and must involve convulsions so pronounced as to render mistake impossible.

CEREBRAL HÆMORRHAGE

unlike the foregoing, is always marked by sudden unconsciousness. The probable age of the patient is a question valuable in diagnosis, as hæmorrhage rarely occurs before forty. The generally complete suspension of intellection, sensation, voluntary and reflex motion, the occurrence of true hemiplegia, the relaxation or paralysis of the sphincters, the stertorous and puffing character of the respiration, the varying temperature, the inequality and insensibility of the pupils, and the frequent lateral deviation of the eyes and head toward the non-paralyzed side, are its most important characteristics.

Those rare cases in which the *Pons Varolii* is the seat of hæmorrhage are most difficult of diagnosis, because the pupils are apt to be equally contracted, the respiration lacks the characteristic stertor, and paralysis is long delayed and often bi-facial. This condition is, in particular, closely simulated by opium narcosis.

MENINGEAL HÆMORRHAGE, although of rare occurrence, is a distinct cause of sudden and profound coma. Its symptoms differ little from those of cerebral hæmorrhage.

A tendency to a remission and recurrence of the attack, the usual appearance of a general motor and sensory paralysis instead of hemiplegia, and the non-impairment of reflex action, are the only differential points we can observe, and whilst these may indicate an involvement of the meninges, it is not easy to determine whether the meningeal lesion is a primary one or whether it is consecutive to a cerebral hæmorrhage by process of invasion.

CEREBRAL EMBOLISM

is another usual cause of sudden unconsciousness. Although possible at any age, yet the *youth* of a patient attacked should be regarded as presumptive evidence in favor of an embolus as opposed to hæmorrhage. The existence of endocarditis or of a valvular heart lesion also offers good grounds for a suspicion of embolism.

A *right-sided* hemiplegia generally appears because the left middle cerebral artery is the usual seat of impaction. This, with a partial loss of sensation, and the general symptoms, as detailed in the summary appended, make up its destinctive features. Erlenmeyer may be quoted as authority for the stated normality of the pupils.

Recovery is usually not long delayed, and the paralysis disappears coincidently with the return of consciousness. A complete absence of paralysis and the occurrence of a variety of epileptiform convulsions have been reported in a few cases.

CEREBRITIS

would not require mention in this connection, but that some exceptional cases are on record, in which coma has been suddenly induced by the rupture of an abscess and escape of its contained pus into the cerebral substance. In these instances slight unilateral convulsions have been noted, and the appearance of the discharge through the auditory, nasal or orbital openings, and the general evidence of inflammatory action, are sufficient diagnostic signs.

SYNCOPE

is so familiar a condition that it is quite unnecessary to enter into its description. Its consideration here is only valuable on account of its relation to other causes of unconsciousness. It is in all cases due to an arrest of function in the cerebral cortex through failure of its arterial blood supply, whether that failure is incident to a general or local anæmia, to sudden failure of the circulation, temporary arrest of the heart's action, or to extensive hæmorrhage from any source.

EPILEPSY

it is equally needless to describe in detail. Its symptoms are well-known, and in ordinary cases easily recognized. After the stage of convulsive action has passed, and coma has deepened, its recognition may, however, be more difficult.

The formerly livid countenance takes on a pale ashen hue; the pulse becomes feeble and irregular; temperature remains high—in some cases as high as 105° F.—the pupils contract, and frothy saliva, sometimes streaked with blood from the bitten tongue, is found upon the lips.

Paralysis is never proper to epilepsy, but it should be remembered that "a paroxysm of epilepsy *may* act as an exciting cause of an apoplectic seizure." A case of this kind is reported in a recent number of the *New York Medical Record.*

CATALEPSY

is the cause of a peculiar form of unconsciousness comparatively easy to differentiate.

Almost invariably peculiar to the female sex; paroxysmal in character; of uncertain duration and constant recurrence; and attended with remarkable muscular rigidity and contortion, it is little apt to offer any possibilities of error.

The feeble, but regular, pulse and respiration; normal temperature; dilated and sensitive pupils; open and tremulous eyelids; anæmic retina, are points worthy of remembrance.

CEREBRAL HYSTERIA, in certain rare cases, is characterized by a sudden loss of consciousness, which may continue for several hours, with slight intervals. It is almost without exception confined to women. It is not accompanied by paralysis, and involves only a partial suspension of intellection, sensation and special sense. The patient can be momentarily aroused, but suffers an immediate relapse. There is little evidence of disturbance of the circulation or respiration. Consciousness is speedily restored on the application of the cold douche.

INSOLATION, or sunstroke, although easy of recognition by means of the presence and prevalence of its exciting cause, is obscure in its pathology. It varies, of course, in duration and intensity. In certain cases cerebral hæmorrhage is induced, when paralysis and other symptoms proper to the latter appear.

The pulse always varies; the rapid and somewhat stertorous respiration is sometimes accompanied by a low moaning sound; the temperature ranges from 104° to 110° F.; the skin is peculiarly harsh and hot, and the pupils contracted and insensible. Vomiting and purging are dangerous symptoms.

URÆMIC POISONING

is a questionable cause of sudden unconsciousness, but certain cases are reported in which the usual prodromata are said to have passed unnoted, until the abrupt occurrence of convulsions and coma.

The convulsions are of the epileptiform type, and liable to remission. The presence of anasarca; the urinous odor of the breath; the dilated, indolent pupils; slow, stertorous respiration of a peculiarly labial character, distinguishable from the guttural sounds characteristic of cerebral hæmorrhage and compression; and the progressively falling temperature, reaching in some cases 91.5° F., furnish sufficient diagnostic evidence. If urinalysis is possible, the discovery of albumen and casts will settle the question of cause.

I cannot overlook the fact that a remarkable difference of opinion exists on the part of Professor Flint, as to the temperature of this condition. He claims that a marked elevation, sometimes ranging as high as 105° F., is observed in these cases. This statement is supported in Ziemssen's Cyclopœdia, where its explanation is attempted by a reference to the epileptiform nature of the convulsions, and the correspondingly high temperature of true epilepsy. But although *epileptiform*, the convulsions are not *epileptic*, and the analogy fails in view of the wide difference in both causes and effects of the two diseases.

Neither can the excessive muscular exertion thus induced be regarded as sufficient cause for this reported elevation. Considering the fact that all other toxic agents which induce coma, among which uræmic poison may properly be classed, invariably occur with *lower* temperature, the above statement appears still more doubtful. In the apparent absence of post-mortem facts to sustain the clinical evidence, I would venture to suggest the occasional occurrence of meningitis as a complication of uræmia, and a possible cause of high temperature, and hence of these conflicting observations.

ASPHXYIA

is properly ranked among comatose conditions of toxic origin, but is too familiar to need anything beyond mention in this connection.

It is well-defined as "a suspension of animation, due to the non-conversion of venous into arterial blood." The indications point to primary disturbance in the lungs rather than the brain.

ALCOHOLIC COMA

would appear, at first thought, to be of all forms of unconsciousness the most easily diagnosticated, and yet no other condition has been so fruitful of mistake, and of injury as the result of error.

A medical verdict of "intoxication" has too often consigned an innocent suffierer from far more serious ills to a course of treatment which involved either irretrievable physical mischief, or a form of moral injury equally impossible of repair.

The odor of alcohol thrown off by the lungs is a valuable aid to diagnosis, but even in the presence of this indisputable evidence of inebriation, it should be remembered that accident incurred while in a drunken state may cause concussion or compression of the brain, or that cerebral hæmorrhage may supervene in this condition. The absence of paralysis, the presence of a complete muscular relaxation and anæsthesia, and the state of the pulse, the respiration and the skin, are all worthy of note, but the condition of the temperature and the pupils furnish the most valuable and distinctive evidence. A low temperature, sometimes markedly low, is not absolutely peculiar to acute alcoholism, as opposed to other forms of unconsciousness, but in none, save that of uræmic poisoning, is there so frequent and so considerable a fall.

Dr. Wm. MacEwen, of Glasgow, Scotland, records a thermic test in fifty cases, noted under many varying circumstances, which shows a downward range of temperature from 97.9° to 93.4° F. These figures represent rectal temperatures, which, according to Wunderlich, should be reckoned from a half to one degree higher than in the axilla. It may therefore be stated that in alcoholic coma the temperature has a latitude of 5° below normal. The same observer claims the demonstration of an absolutely pathognomonic sign in the condition of the pupils, which, as far as my observation and inquiry have extended, has proved a reliable aid in the diagnosis of doubtful cases and the exclusion of any coincident injury or disease.

In alcololic coma *undisturbed*, he states that the pupils are *invariably contracted*, but that on the application of any external stimulus, sufficient to partially arouse the patient, they *at once dilate*, again relapsing into a contracted state as unconsciousness re-deepens. Dr. MacEwen reports the observation of fifty cases, of which number forty-eight answered perfectly to the test, contraction recurring in from five to thirty minutes after stimulation. The two exceptions to this rule proved, on subsequent inquiry, to have formerly suffered with disease of the iris, which had resulted in complete fixity of the pupils. Since the publication of these items I have endeavored, as opportunity offered, to test their accuracy, and in some eight cases I have noted, the behavior of the pupils has been in perfect accord with the facts stated. This alternation is, I believe, characteristic of no other comatose condition, and is therefore, if supported by further investigation, an invaluable diagnostic sign.

OPIUM NARCOSIS

is so rapidly fatal in its course that it especially demands prompt recognition. In the early stages of its action the patient can be momentarily aroused. The odor of opium on the breath can usually be detected. The complete muscular relaxation, the suppression of all secretions except that of the skin which is very profuse, the usually persistent vomiting, the markedly contracted and insensible pupils, and slightly reduced temperature, with other general symptoms, should be born in mind.

The symptoms attending the narcosis of other soporific poisons are similar to the above. In the case of chloral, dilatation instead of contraction of the pupils is observed when poisoning results from the slow cumulative action of the drug.

In closing the discussion of these causes of sudden unconsciousness, let me refer briefly to those surgical conditions known as

CEREBRAL CONCUSSION, CONTUSION AND COMPRESSION.

Any attempt to differentiate their peculiar symptoms is necessarily limited by the fact that the occurrence of either of these distinctive forms of injury uncomplicated with one or both of the others is exceedingly rare. Indeed, Dr. Bryant explicitly stated that "consciousness and contusion of the brain are *always* associated in fatal cases," and the Reports of Guy's Hospital, London, for fifteen years, record "no case of death from concussion without change of brain structure."

Compression doubtless often happens alone. The possibility of an apoplectic or epileptic seizure occurring in a position likely to provoke a suspicion of accident, or even inducing a fall, should always be remembered.

*Cerebral Concussion*, pure and simple, is believed by many surgeons to be little more than a momentary disturbance, and certainly cases on record in which coma has persisted for hours or even days, can hardly be regarded as uncomplicated. The loss of consciousness resulting, is usually immediate and complete.

*Cerebral Contusion*, is defined to be "a sudden and violent attrition of the brain substance, attended with more or less laceration and effusion of blood," generally in the form of minute hæmorrhagic points. It does not necessarily involve injury, actual or apparent, to the calvaria. Its symptoms as apart from those of concussion and compression are not well differentiated.

*Cerebral Compression*, of traumatic origin, may depend upon the pressure of fragments of the outer or inner tables of the skull, or of a blood-clot formed beneath the seat of injury.

A short interval usually elapses between the infliction of the injury and the appearance of the profound unconsciousness which follows. The general identity of its symptoms with those attendant upon cerebral hæmorrhage in its idiopathic form, indicates that the results are substantially the same in the two cases.

For the general symptoms observable in these surgical cases, as well as in the other foregoing morbid conditions, I would refer to the summarized table appended to this sketch, which offers a readier means of rapid comparison than it would be possible to give in any other form.

It cannot but be observed that, in many cases, the means of differentiation we possess are at present very limited, and even those most clearly defined are often clouded by varying complications.

There only remains to allude briefly to the *modus operandi* of the causes which result in this general effect of unconsciousness, and on this important but obscure question I shall venture but a few simple suggestions.

The post-mortem evidence recorded throws but little light upon the working of these causes, but, applying the few facts thus obtained to the clinical history we possess, the indications seem to point strongly to *one* pathological condition necessary to the production of unconsciousness in any of its forms, viz.: a deficiency of arterial or oxygenated blood in the vessels of the brain. If this point be well taken, we have at once an explanation of the difficulty we meet in attempting to differentiate these conditions, in the fact that, though induced by exciting causes of widely varying nature, in many varying ways, and in greatly varying degree, the ultimate result, manifested in the loss of consciousness, is in each and all the same.

Thus, in syncope, probably the simplest form of unconsciousness, we find an anæmia of the cerebral cortex, whether caused by arrest of the general circulation, by profuse hæmorrhage from other parts, or by a chronic anæmic condition. In catalepsy and cerebral hysteria, the symptoms point to the same result, dependent upon deranged nervous function, probably manifesting itself through the vaso-motor system by inducing a tonic contraction of the cerebral arterioles. In concussion of the brain, the theory of vascular paralysis affecting the vessels of the cortical portion of the cerebrum and producing arterial anæmia, with consequent venous stasis, offers the best explanation of the phenomena observed; and seeing that we have no knowledge of the long continuance of such vascular paralysis, it accords well with the opinion that true concussion is but of short duration.

Insolation furnishes us with an instance of more complicated action, but it is probable, as Niemeyer observes, that the febrile condition of the blood, caused by the excessive heat and exposure, involves the loss of its oxygenating power.

Epilepsy has hitherto been very obscure in its pathology, but leading neurologists support the theory that the coma is due to general cerebral anæmia, while they refer the attendant convulsions to the excitation of a "convulsive center," by means of a local hyperæmia or congestion. This question doubtless requires further demonstration.

In the toxic conditions we have reviewed, we can distinctly trace the disturbance of cerebral function to the influence of the toxic agent, either directly upon the blood, as in uræmia and asphyxia; upon the heart and vessels through the agency of the nervous system, as in opium narcosis; or upon both, as in alcoholic intoxication.

By whatever mode of action, the results seem to be in each instance the same—that is, either the destruction or impairment of the oxygenating power of the blood, or an interference with the circulation in the cerebral substance.

In cerebral and meningeal hæmorrhage, and in traumatic compression, a similar end is reached by means of mere mechanical pressure.

Dr. Bryant observes that "unconsciousness in these cases, whether from injury or hæmorrhage, appears as soon as commencing intra-cerebral pressure impinges upon the capillaries of the cortex," and it is probable that it ensues even before that point is reached.

The rupture of an abscess into the brain substance has the same mechanical effect, and embolism, obstructing the cerebral arteries, induces partial pressure, and causes a more circumscribed anæmia, which, in its turn, accounts for the slighter and more transient symptoms.

The uncertainty which evidently surrounds this question in the minds of scientific observers, and leaves room for these imperfect suggestions, offers a wide field for patient investigation and research.

THE DIFFERENTIAL DIAGNOSIS OF

| | CAUSES. | PULSE. | RESPIRATION. | TEMPERATURE. | PUPILS. |
|---|---|---|---|---|---|
| 1 | CEREBRAL CONGESTION. | Slow and full. | Slow and labored. *Not* stertorous. | Above normal. | Contracted with feeble reaction. |
| 2 | CEREBRAL HÆMORRHAGE. | Slow, full, often irregular. | Slow, labored and stertorous. | 1. Below Normal. 2. Later regains normal. 3. Still later rises rapidly. (105–7°) | Variably unequal, insensible to light. |
| 3 | MENINGEAL HÆMORRHAGE. | Slow, full, and irregular. | Slow and stertorous. | Variable. | Variable, insensible to light. |
| 4 | CEREBRAL EMBOLISM. | Slow, weak and small. | Slow and full. | Slightly lowered. | Normal. |
| 5 | CEREBRITIS. | Rapid and irregular. | Shallow and irregular. | Higher than normal. | Unchanged. |
| 6 | SYNCOPE. | Rapid and thready or extinct. | Feeble and quiet. | Below Normal. | Dilated. |
| 7 | EPILEPSY. | Rapid, feeble and irregular. | Difficult, irregular, often gasping. | High to (105° F). | At first dilated, later contracted. Insensible to light. |
| 8 | CATALEPSY | Normal rate, but feeble. | Slow and regular. | Normal. | Dilated and very sensitive to light. |
| 9 | CEREBRAL HYSTERIA. | Small, feeble, but regular. | Rapid and quiet. | Normal. | Unchanged. |
| 10 | INSOLATION. | At first, slow and full. Later, rapid, feeble and frequent. | Rapid and slightly stertorous. | Very high. (104°-10° F.) | Contracted. Insensible to light, |
| 11 | URÆMIA. | Slow, feeble and irregular. | Slow and labored, with peculiar stertor. | Progressively lowered. | Dilated and indolent. |
| 12 | ASPHYXIA. | Slow and feeble. | Slow, feeble and gasping. | Lowered. | Variable, usually contracted. |
| 13 | ALCOHOLIC INTOXICATION. | Small, feeble and frequent. | Slow and labored. Sometimes stertor's. | Much lowered. | Contracted Dilate under stimulation and again relapse. |
| 14 | OPIUM NARCOSIS. | Small, feeble and irregular. | At first frequent. Later superficial and slow. | Normal or slightly lowered. | Much contracted, insensible to light. |
| 15 | CEREBRAL CONCUSSION. | Slow, feeble and intermittent. | Feeble, sighing or almost extinct. | Below normal. | Unequal React feebly |
| 16 | CEREBRAL CONTUSION. | Slow and feeble. | Slow but quiet. | Normal or Slightly elevated. | Contracted. Insensible to light. |
| 17 | CEREBRAL COMPRESSION. | Slow, soft and irregular. | Slow, labored and stertorous. | Variable, usually increased. | Dilated or unequal. Insensible to light. |

CAUSES OF SUDDEN UNCONSCIOUSNESS.

| MOTOR POWER. | INTELLECTION. | SENSATION. | SPECIAL SENSES. | COUNTENANCE. | MISCELLANEOUS. |
|---|---|---|---|---|---|
| Partial paralysis. Reflexes impaired. | Wholly or in part lost. | Impaired. | Partially impaired. | Flushed or Cyanosed | Venous distention of face and neck. Retinal engorgement |
| Hemiplegia. Reflex action abolished. | Completely suspended. | Entirely lost. | Impaired. | Variable. | Sphincters relaxed or paralyzed. Deglu. tition impaired. Ptosis frequent. |
| Paralysis more or less ge[illegible]ral. Reflexes unimpaired. | Suspended. | Anæsthesia accompanying paralysis. | Somewhat impaired. | Variable. | Sphincters relaxed. Vomiting usual. |
| Hemiplegia, usually right-sided. Reflex action normal. | Transiently suspended. | Partially lost. | Normal. | Pale. | |
| Muscular relaxation. Slight convulsions. Reflex action suspended. | Suspended. | Hyperæsthesia. | Unimpaired. | Variable. | General evidences of inflammation. |
| Muscles relaxed. Reflexes unimpaired | Suspended. | Impaired. | Impaired. | Very Pallid. | Cardiac and venous murmurs. |
| Convulsions. Reflex action somewhat impaired. | Suspended. | Lost. | Unimpaired. | Pale. Ashen hue. | Eyelids tremulous. Eyeballs prominent. Tongue bitten. |
| Muscular rigidity. Reflexes disturbed. | Suspended. | Impaired. | Partially Impaired. | Pallid. | Retinal anæmia. Eyelids tremulous. |
| Normal. | Partially Suspended. | Blunted. | Somewhat impaired. | Natural. | Spasmodic movements of eyes and eyelids. |
| Muscular relaxation. Reflex action feeble. | Completely Suspended. | Impaired. | Impaired. | Variable. | Skin harsh and hot Vomiting and purging. |
| Epileptiform convulsions. Reflex action feeble. | Suspended. | Impaired. | Impaired. | Pallid. | Anasarca. Urinous odor of breath. Albumen and casts in urine. |
| Convulsions. Reflex action impaired. | Suspended. | Impaired. | Disturbed. | Turgid. | |
| Muscular relaxation. Reflex movements suspended. | Suspended. | Complete Anæsthesia. | Impaired. | Variable. | Odor of alcohol on the breath. |
| Complete muscular relaxation. Reflexes abolished. Convuls'ns in morphia poison'g. | Suspended. | Impaired. | Impaired. | Pale and shrunken. Sometimes livid. | Odor of Opium on breath. Vomiting. Secretions arrested, except that of skin. |
| Muscles weak and flaccid. Reflexes impaired. | Suspended. | Entirely lost. | Act feebly. | Very pallid. | Surface cold. Vomiting frequent. Paralysis of bladder and sphincters. |
| Muscular rigidity. Slight facial spasms. Paralysis of eyelids. Reflexes impaired. | Suspended. | Impaired. | Impaired. | Pale | Sphincters relaxed. |
| Hemiplegia. Reflexes abolished. | Suspended. | Entirely lost. | In abeyance. | Very pale. | Deglutition impossible. Sphincters paralyzed. Retention of urine. |

